# Mitochondrial DNA Heteroplasmy and Purifying Selection in the Mammalian Female Germ Line

**DOI:** 10.1111/dgd.12420

**Published:** 2018-01-24

**Authors:** Stephen P. Burr, Mikael Pezet, Patrick F. Chinnery

**Affiliations:** ^1^ MRC Mitochondrial Biology Unit Department of Clinical Neurosciences University of Cambridge Cambridge UK

## Abstract

Inherited mutations in the mitochondrial (mt)DNA are a major cause of human disease, with approximately 1 in 5000 people affected by one of the hundreds of identified pathogenic mtDNA point mutations or deletions. Due to the severe, and often untreatable, symptoms of many mitochondrial diseases, identifying how these mutations are inherited from one generation to the next has been an area of intense research in recent years. Despite large advances in our understanding of this complex process, many questions remain unanswered, with one of the most hotly debated being whether or not purifying selection acts against pathogenic mutations during germline development.

## Graphical abstract

In this review, we synthesize current knowledge regarding the germline transmission of mtDNA mutations and examine the evidence for and against purifying selection during this process, focusing primarily on data from humans and other mammals, but drawing on additional species where relevant.

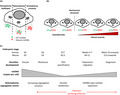

## Mitochondrial DNA mutation: homoplasmy versus heteroplasmy

**Figure 1:**
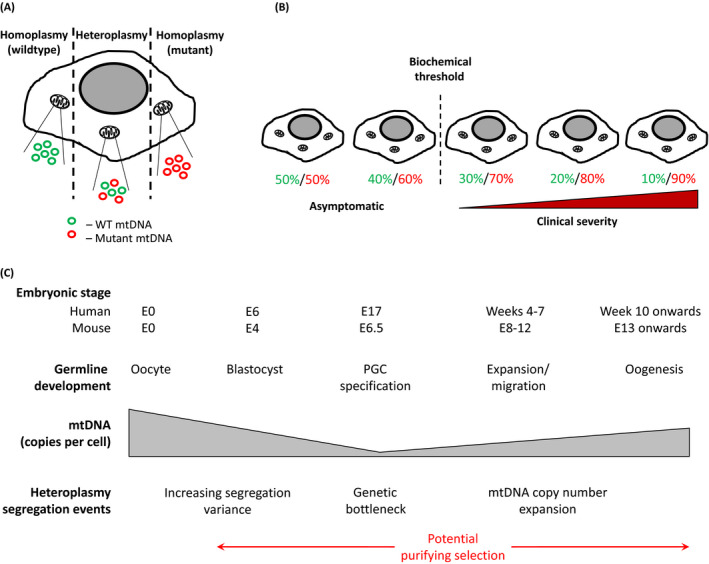
mtDNA heteroplasmy and its transmission through the female germline. (A) Each cell contains multiple mtDNA molecules, a mutation in the mtDNA (red circles) is termed homoplasmic if all copies carry the mutation, or heteroplasmic if only a proportion carry it. (B) In heteroplasmic cells, as the burden of a pathogenic mutation (red figures) increases compared to wildtype mtDNA (green figures), a biochemical threshold is reached. Beyond this point the cell can no longer compensate for the mutation and a respiratory chain defect develops. The severity of this defect tends to worsen with further increase in the levels of mutant mtDNA. (C) Schematic representation of female germline development during embryogenesis, and associated events determining the differential segregation of heteroplasmic mtDNA variants. Whilst the contribution of purifying selection to this process is controversial, such mechanisms could be active from the initial decrease in cellular mtDNA copy number leading up to the genetic bottleneck, during PGC specification and development and through oogenesis into adult life.

The mammalian mitochondrial (mt)DNA genome consists of a circular, double‐stranded loop of DNA varying from 15 000 to 17 000 bp in length depending on the species, with the human mtDNA sequence containing 16 569 bp (Chinnery & Hudson 2013). The mtDNA contains 37 genes, encoding 13 subunits of the electron transport chain (ETC), which contribute to the production of energy in the cell via oxidative phosphorylation (OXPHOS); and 24 RNA, comprising 22 tRNA and two ribosomal RNA (16S RNA and 12S RNA), required for the transcription and translation of mtDNA‐encoded proteins (Chinnery & Hudson 2013). Unlike the nuclear genome, mammalian mtDNA is inherited uniparentally, solely via the maternal line, and does not undergo recombination (Hagstrom *et al*. 2014). mtDNA is present in multiple copies within each cell, and copy number varies from 100 to 10 000 copies, adapting to the cellular needs in a tissue‐specific manner (Chinnery & Hudson 2013). mtDNA has a much higher mutation rate than the nuclear genome, possibly due to the close proximity of mtDNA to mutagenic reactive oxygen species (ROS) (Lagouge & Larsson 2013), continuous replication of mtDNA in post‐mitotic cells, with an error rate several orders of magnitude higher than in the nucleus (Johnson & Johnson 2001), or a less extensive array of DNA repair mechanisms (Kazak *et al*. 2012; Scheibye‐Knudsen *et al*. 2015) compared with the nucleus. Novel mtDNA mutations invariably lead to a heterogenic state termed “heteroplasmy”, where wild‐type molecules coexist with mutated mtDNA molecules in the same cell, in contrast to the normal state of “homoplasmy”, where all copies of mtDNA present in the cell share the same sequence (Fig. 1A).

## mtDNA mutations and purifying selection throughout evolution

In 1964, Muller postulated that asexual inheritance of DNA without recombination should lead to the accumulation and fixation of deleterious mutations. In the absence of purifying selection to remove these mutations, this process will ultimately result in mutational meltdown of the genome, a hypothesis known as “Muller's ratchet” (Muller 1964). Evidence of this predicted build‐up of mutations in mtDNA can be seen in phylogenetic data from human lineages. In theory, all human mtDNA can be traced back to a single woman, the so‐called “Mitochondrial Eve”, who lived in Africa approximately 200 000 years ago (Cann *et al*. 1987). Since that time the mtDNA genome has been constantly acquiring point mutations, resulting in the development of a number of discrete haplogroups, each defined by a specific subset of variants (Torroni *et al*. 1997; Wallace *et al*. 1999). It is important to note that each of these variants must first have existed as a heteroplasmic mutation, before becoming fixed homoplasmic polymorphisms, and some mitochondrial diseases, such as Leber hereditary optic neuropathy, are primarily caused by homoplasmic mutations (Yu‐Wai‐Man *et al*. 2002). Despite the presence of fixed variants in the mitochondrial genome, which in rare cases can be pathogenic, many mtDNA‐related mitochondrial diseases are caused by heteroplasmic point mutations in the mtDNA coding sequence, which disrupt ETC activity and lead to mitochondrial dysfunction (Stewart & Chinnery 2015). However, it is important to note that heteroplasmic mutations can either be inherited or acquired through de novo somatic mutation during embryogenesis or postnatal development. Although homoplasmic variants may be selected against at the population level (Stewart *et al*. 2008a), heteroplasmic mutations can additionally be subject to purifying selection within individuals and during transmission from generation to generation (Li *et al*. 2016), allowing them to be removed from the population before becoming fixed in the mtDNA sequence. The fact that relatively few highly deleterious variants have become fixed in the mtDNA genome, in spite of its elevated mutation rate, suggests that purifying selection against such mutations must be active, preventing the rapid advancement of Muller's ratchet (Rand & Kann 1996, 1998; Elson *et al*. 2004; Rand 2008).

The efficiency of purifying selection cannot be 100%, because deleterious heteroplasmies are found relatively commonly in the general population (Elliott *et al*. 2008; Payne *et al*. 2013), and novel heteroplasmies have been repeatedly allowed to fix in the mtDNA genome throughout evolution, thus “escaping” selection altogether. Additionally, the mutations that “escape” selection are not merely neutral variants, as many of the haplogroup polymorphisms found in human mtDNA are non‐synonymous and potentially affect mitochondrial function (Kazuno *et al*. 2006; Pello *et al*. 2008; Gomez‐Duran *et al*. 2010). It has been suggested that positive selection of advantageous mtDNA variants may explain the presence of some haplogroup polymorphisms. The founding mutations of several haplogroups appear to be linked to major migratory events during human colonization of the globe, and might have allowed survival in colder climates as our ancestors moved out of Africa (Coskun *et al*. 2003; Mishmar *et al*. 2003; Ruiz‐Pesini *et al*. 2004); however, this theory is controversial and not universally accepted (Elson *et al*. 2004; Kivisild *et al*. 2006). Whilst it is tempting to envisage a simple model where advantageous heteroplasmies are retained and become fixed, and deleterious variants are purged, this is clearly not the case. Some haplogroup‐specific variants have been shown to predispose to certain diseases (Hudson *et al*. 2013, 2014; Jimenez‐Sousa *et al*. 2015), with some affecting individuals during reproductive life. Therefore, a balance clearly exists between the appearance of novel heteroplasmic mtDNA mutations and subsequent selection against the most pathogenic of these variants.

## Potential modes of purifying selection

There are a number of levels at which this process of purifying selection could occur (Rand 2001). In addition to the aforementioned selection at the population level, mtDNA mutations may be selected against at the level of individual organisms, via classical Darwinian selection, with higher levels of mutation resulting in reduced fitness and less chance of passing the mutation to the subsequent generation. At the cellular level, variations in mutational load between cells will result in selection against those cells that are least fit (i.e. carry the highest levels of mutation) (Rajasimha *et al*. 2008). This variation may originate either when cells divide, or during replication of mtDNA genomes in post‐mitotic cells (Chinnery & Samuels 1999). Because mtDNA replication is an ongoing process, even in non‐dividing cells, segregation is active in all tissues throughout life (Burgstaller *et al*. 2014), and purifying selection may therefore play an important role at all stages. Finally, selection may also occur at the subcellular level, potentially through preferential replication of a subset of mtDNA within the cell prior to division (Blok *et al*. 1997).

When considering the transmission of heteroplasmic mtDNA mutations, the development of the maternal germline is a unique and intriguing step, as it represents the point at which the single cell and the whole organism converge. Therefore, any selective mechanisms active on the mtDNA at this stage have huge potential to influence the integrity of the mtDNA genome in subsequent generations. This is one of the reasons that germline inheritance of heteroplasmy has been such an intense area of research in recent years, and perhaps one of the most important advances in this field has been the development of the germline genetic bottleneck theory of mtDNA inheritance.

## Maternal inheritance of mtDNA heteroplasmy: the genetic bottleneck theory

In patients carrying potentially deleterious heteroplasmic mtDNA mutations, the severity of disease symptoms tends to correlate with the level of heteroplasmy, and if a certain biochemical threshold is reached the individual will develop pathogenic phenotypes (Fig. 1B) (Chinnery *et al*. 1997; DiMauro & Schon 2001). Very low level mtDNA heteroplasmy appears to be universal in the human population (Payne *et al*. 2013), and more than 1:200 healthy live births carry a point mutation present at a more than 1% heteroplasmy level (Elliott *et al*. 2008). Because mtDNA is strictly maternally inherited (Hutchison *et al*. 1974; Case & Wallace 1981; Pyle *et al*. 2015) and the heteroplasmy level transmitted from a mother to her offspring varies significantly (Larsson *et al*. 1992; Blok *et al*. 1997), providing prognostic advise to healthy women carrying a pathogenic mtDNA mutation is currently very challenging (Poulton *et al*. 2010; Chinnery *et al*. 2014).

Shifts in heteroplasmy between a mother and her offspring were first observed in Holstein cows harboring two mitochondrial genotypes, distinguished by a single point mutation. In a single maternal lineage, significant heteroplasmy shifts were observed within a few generations (Hauswirth & Laipis 1982). Hauswirth and Laipis suggested that such variation could be due to a dramatic reduction of the mtDNA copy number during oogenesis, resulting in an increased likelihood of heteroplasmy segregation by genetic drift: the so‐called “genetic bottleneck” theory. This change in copy number was later evaluated in mice and shown to decrease from 100 000 copies in the fertilized oocyte to just 200 copies in the PGC, with an estimated 40 mitochondria per cell containing five mtDNA molecules each at this stage (Fig. 1C) (Nass 1966; Nogawa *et al*. 1988; Jenuth *et al*. 1996). This mtDNA bottleneck effect in the PGC has since been corroborated by computational simulation and quantitative PCR measurements at the single cell level (Cree *et al*. 2008; Wai *et al*. 2008). Nonetheless, this theory remains controversial because additional work utilizing the same animal model failed to show such a drastic decrease in the mtDNA copy number, with the lowest mtDNA content estimated to be approximately 1500 copies per cell (Cao *et al*. 2007, 2009).

The mtDNA germline genetic bottleneck seems to be present in other vertebrate species. In zebrafish, Otten *et al*. measured the mtDNA copy number during embryogenesis, from fertilized oocyte to PGC. They observed a marked decrease from 2.0 × 10^7^ copies per cell in the fertilized oocyte to 170 copies per cell in the PGC (Otten *et al*. 2016), suggesting a strong bottleneck effect, similar to that observed in mice. Measurements of mtDNA copy number have also been performed at later stages during oogenesis in mammalian species. In sheep, Cotterill *et al*. compared the mtDNA content at the primordial follicle stage to the metaphase II oocyte stage. They observed an increase from 605 copies per cell in the primordial follicle to 7.5 × 10^5^ copies per cell in mature oocytes, suggesting a bottleneck event following the primordial follicle stage (Cotterill *et al*. 2013). It is interesting to note that, even though the primordial follicle stage occurs later than the PGC specification during the embryogenesis, the mtDNA copy number observed in this study remains lower than the measurements performed by Cao *et al*. (2007).

Thus, numerous independent studies point toward the genetic sampling event during oogenesis that was first proposed by Hauswirth and Laipis (1982). The reduction of mtDNA copy number is predicted to lead to a shift in heteroplasmy that can happen within a few generations (Hauswirth & Laipis 1982; Jenuth *et al*. 1996; Cree *et al*. 2008). However, over 30 years since this hypothesis was first put forward, the exact timing of the mtDNA copy number decrease during oogenesis and its impact on heteroplasmy shifts between generations remains elusive. Wai *et al*. (2008) did not observe significant differences between the level of heteroplasmy of female mice and the PGC of their progeny, as had previously been suggested (Hauswirth & Laipis 1982; Jenuth *et al*. 1996). Instead, they found that shifts in heteroplasmy appeared to occur during postnatal folliculogenesis. The authors suggested that this “late” genetic bottleneck was due to the replication of a subpopulation of mtDNA molecules during the maturation phase of the follicles (Wai *et al*. 2008). The observation by Cao *et al*. that mouse PGC contained higher mtDNA copy number than previously predicted (1500 copies, compared with the 200 copies reported by Cree *et al*.) led to a third bottleneck theory, involving unequal segregation of the mtDNA molecules during cell division, rather than a drastic reduction in copy number (Cao *et al*. 2007; Cree *et al*. 2008). However, direct evidence supporting this mechanism is lacking. More recently, Freyer *et al*. (2012) investigated the timing of heteroplasmy changes of novel mtDNA mutations by backcrossing a female mouse carrying a *PolgA exo* ^−^ mutation (also called the mtDNA mutator mouse (Trifunovic *et al*. 2004)) with wild‐type males. Using this method, they obtained a maternal lineage carrying an m.3875delC in the tRNA^Met^ gene. Measurement of heteroplasmy levels in maternal cells, embryonic PGC and oocytes/soma of the offspring suggested a shift of heteroplasmy occurring during the early stages of PGC development, lending further support to the “early” bottleneck hypothesis (Freyer *et al*. 2012).

These contrasting observations highlight the current ambiguity concerning the timing and mechanism of the mitochondrial germline genetic bottleneck. The differences may, in part, reflect true biological differences between different strains and species. However, an alternative explanation is that technical differences, due to the measurement of low quantities of mtDNA in single cells, or the precise timing of the observations during development, could contribute to the different results reported. However, whatever the precise underlying process may be, the unpredictable shifts in heteroplasmy transmitted from mothers carrying pathogenic mutations to their children continue to make this area of research highly relevant. One key question that remains unanswered is whether or not the germline genetic bottleneck plays a role in selection against deleterious mtDNA heteroplasmies during transmission of the mtDNA from mother to offspring (Fig. 1C).

## Evidence for and against purifying selection in mouse pedigrees

Much work on germline transmission of mtDNA has utilized invertebrate model organisms, including *Drosophila* (Hill *et al*. 2014; Ma *et al*. 2014) and *Caenorhabditis elegans* (Wernick *et al*. 2016). However, whilst these species provide tractable models for such studies, they are far removed from humans in evolutionary terms, and so here we focus on data from a range of studies in mammals that have attempted to address the role (if any) of purifying selection in inheritance of mtDNA heteroplasmies.

Transmission of mtDNA has been studied in a number of mammalian species, including cows, sheep and zebrafish, but many of the studies to date have been conducted in mice. The inability to genetically manipulate mammalian mtDNA has made the study of pathogenic heteroplasmies in mouse models challenging. Early studies relied on fusing cytoplasts from two different mouse strains to generate conplastic heteroplasmic animals carrying two separate mtDNA genotypes. Jenuth *et al*. (1996) studied segregation of heteroplasmy in conplastic NZB/BALBc mice, concluding that the variance of this non‐pathogenic heteroplasmy in germline cells was due to random genetic drift. Using a similar approach, Meirelles and Smith (1997) generated a conplastic NZB/C57Bl6 mouse strain and saw evidence of stable heteroplasmy across generations, contrasting the variance seen by Jenuth *et al*. (1997), and inconsistent with the existing bottleneck theories. Interestingly, Sharpley *et al*. (2012) found that mixing of mtDNA from the NZB and 129S6 strains resulted in animals that developed pathogenic phenotypes, a phenomenon also seen by Acton *et al*. (2007) in NZB/BALBc conplastic mice. Sharpley *et al*. (2012) showed that heteroplasmy in NZB/129S6 mice segregated rapidly towards 129S6 homoplasmy over successive generations, suggesting active selection against transmission of NZB mtDNA. These contrasting results highlight the complex nature of mtDNA inheritance, and suggest that pathogenic and non‐pathogenic heteroplasmies may segregate differently, and may be influenced by the nuclear genetic background.

More recently, several groups have been able to generate mice harboring deleterious heteroplasmic mtDNA mutations, allowing more detailed investigation of how pathogenic variants are transmitted through the germline. Fan *et al*. (2008) used a complex approach involving cytoplast fusion of mouse ES cells, and subsequent injection into C57Bl/6 blastocysts, to introduce two heteroplasmic mutations into the mtDNA of a single female mouse. One mutation was a highly deleterious frame‐shift in the ND6 gene, and the second a less severe missense mutation in the cytochrome c oxidase I (COI) gene. Analysis of heteroplasmy levels in subsequent generations revealed rapid elimination of the severe ND6 mutant within four generations, whilst the milder COI mutant persisted, despite causing myopathy and cardiomyopathy in the mice that carried it (Fan *et al*. 2008). These results suggest that purifying selection may act rapidly against severe pathogenic mutations, whilst allowing less severe variants to persist in the population. In a separate study, Stewart *et al*. (2008b) utilized the *PolgA exo* ^−^ mtDNA mutator mouse model to introduce random mutations into the maternal mitochondrial genome and studied their transmission. Using this approach, they also found evidence that non‐synonymous (i.e. possibly pathogenic) mutations in protein coding sequences are rapidly purged over just a few generations, lending further support to the hypothesis that deleterious variants are subject to purifying selection (Stewart *et al*. 2008b).

The point at which purifying selection occurs in the mouse is currently not well defined. In the aforementioned study by Freyer *et al*. (2012), segregation of the tRNA^Met^ m.3875delC mutation was seen early in PGC development, consistent with the presence of a germline genetic bottleneck. However, evidence of purifying selection against high levels of mutation was only seen in postnatal tissues, and not during germline development (Freyer *et al*. 2012). A similar study in mice carrying a point mutation in the tRNA^Ala^ gene also identified selection against high levels of heteroplasmy in subsequent generations, but there was no concurrent increase in embryonic death, again suggesting that the selection takes place at the cellular/organellar level after birth (Kauppila *et al*. 2016). Despite these findings, there is currently very little data available on the transmission of pathogenic mtDNA mutations through the mouse germline, and further work will be required to fully understand the dynamics of purifying selection in this context.

## Evidence for and against purifying selection in human pedigrees

Understanding heteroplasmy transmission in the human germline presents an even greater challenge than that faced in the mouse. This is partly due to the difficulty of obtaining embryonic tissues for analysis and the ethical constraints that must be considered when dealing with such sensitive material, but also because of the inherent problem of ascertainment bias when obtaining pedigree data from an affected proband (Wilson *et al*. 2016). Consequently, the number of studies in this area is few, although a number of groups have managed to make some progress in this challenging field. Monnot *et al*. (2011), who analyzed embryonic tissues from nine heteroplasmic females carrying the common m.3243A>G mutation, responsible for mitochondrial encephalomyopathy, lactic acidosis and stroke‐like episodes syndrome, found that segregation appears to be governed by random genetic drift during early embryonic development. Similar results were seen in oocytes and embryos for both the m.3243A>G mutation (Brown *et al*. 2001) and the m.8993T>G neuropathy, ataxia and retinitis pigmentosa mutation in (Blok *et al*. 1997; Steffann *et al*. 2006, 2007), suggesting that these variants are not subject to purifying selection. However, it must be noted that drawing firm conclusions from small‐scale studies such as these is difficult because reliable statistics require many independent observations (Wonnapinij *et al*. 2010). More recently, an analysis of human pedigrees transmitting a number of common pathogenic heteroplasmies found that, although the rate of segregation appears to vary between different mutations, there was no evidence of selection from mother to offspring (Wilson *et al*. 2016).

Although the above studies all seem to argue against purifying selection acting on mtDNA variants during germline development, these findings contrast with other similar studies; Rebolledo‐Jaramillo *et al*. (2014) sequenced mtDNA from 39 healthy mother–child pairs, and found that most carried one or more low‐level heteroplasmies, some of which were disease associated. Analysis of these point mutations showed reduced transmission of non‐synonymous compared with synonymous mutations, suggesting that potentially pathogenic variants are selected against. Similarly, Li *et al*. (2016) also identified selection against novel deleterious mtDNA heteroplasmies in a large dataset obtained from the Genomes of the Netherlands project. Furthermore, *in vivo* data from recent analysis of oocytes from nine healthy women found evidence of selection against potentially pathogenic mtDNA variants during oogenesis, occurring between the expulsion of the first and second polar bodies (De Fanti *et al*. 2017). Finally, very recent data from Floros *et al*. (2018), obtained from early‐gestation human embryos (Carnegie stages 12–21), suggests that non‐synonymous mtDNA mutations are indeed subject to purifying selection during PGC development. These conflicting results highlight the fact that our understanding of the complex mechanisms underpinning mtDNA transmission is far from complete, and much work remains to be done to fully elucidate this key process.

## Proposed mechanisms by which purifying selection may occur

The mechanism(s) controlling purifying selection in the mammalian germline are not clear, and there is currently much debate over whether this occurs purely by random genetic drift or by active selection. Whilst this issue remains unresolved, it is quite possible that the underlying processes vary depending on the specific mutation and the level of heteroplasmy present, with the additional complications raised by the different nuclear genetic backgrounds.

Despite the evolutionary differences between mammals and invertebrates alluded to previously, two recent studies in model organisms have shed light on potential mechanisms involved in the transmission of heteroplasmic mtDNA mutations and warrant mention here as potential pathways of interest to investigate further in mammals. Hill *et al*. (2014) generated a mutant *Drosophila* line carrying a temperature‐sensitive mtDNA heteroplasmy in the COI gene. During oogenesis, they found that wild‐type mtDNA from “healthy” mitochondria was preferentially amplified over those containing high levels of the mutant version, suggesting that selection against heteroplasmy is dependent upon “mitochondrial fitness”. In contrast, Lin *et al*. (2016) engineered a *C. elegans* strain harboring an mtDNA deletion heteroplasmy, and identified that the mitochondrial unfolded protein response (UPR^mt^) plays an important role in maintenance of this deleterious mutation. It remains to be seen whether either of these proposed mechanisms plays any role in mammalian cells, although recent data from Pezet and colleagues has failed to identify UPR^mt^ activation in human cybrids carrying a heteroplasmic mtDNA deletion, suggesting that this mechanism is not involved in maintenance of heteroplasmy in human cell lines (Mikael Pezet, unpub. data, 2017).

In mice, Battersby and Shoubridge (2001) attempted to identify the mechanism underlying preferential segregation of NZB mtDNA in liver tissue of NZB/BALBc conplastic animals (Jenuth *et al*. 1997). They concluded that selection against BALBc mtDNA was not due to growth defects or altered OXPHOS capacity, but instead likely depended upon factors involved in mtDNA maintenance (Battersby & Shoubridge 2001). Subsequently, Moreno‐Loshuertos *et al*. (2006) reported that NZB/BALBc heteroplasmy does result in altered OXPHOS efficiency due to an SNP in the tRNA^Arg^ gene, the effects of which are masked by compensatory mechanisms triggered by upregulation of ROS production. However, these findings were refuted by Battersby and Shoubridge (2007), and Freyer *et al*. (2012) have since published further evidence that selection against heteroplasmy is not dependent upon OXPHOS function. Thus, a definitive mechanism for this selection remains elusive in this context. In the same NZB/BALBc model, Jokinen *et al*. (2015) studied the preferential selection for BALBc mtDNA in hematopoietic cells and found that the tail‐anchored, ER‐resident GTPases Gimap3 and Gimap5 play a critical role in mtDNA segregation, suggesting that coordination of organelle interactions may be involved in modulating mtDNA segregation and selection.

Studies investigating these selective mechanisms in humans are similarly sparse. Blok *et al*. (1997) suggested preferential amplification of a subset of mitochondrial genomes, possibly similar to that seen in *Drosophila* (Hill *et al*. 2014), to explain skewed segregation of the m.8993T>G mutation in human oocytes, but there is no empirical human data to support this theory. More recently, Ling *et al*. (2016) found that fibroblasts carrying the m.3243A>G mutation showed increased segregation towards homoplasmy following treatment with ROS, and suggested that this was due to formation of mtDNA concatemers that allow amplification of multiple identical mtDNA copies as a single unit. Whether this mechanism has any role in mtDNA segregation during germline development is currently not clear.

## Current tools for studying purifying selection in the germline

Although we currently understand very little about the selective mechanisms active in the female germline, recent advances in both murine and human reproductive biology have provided important new tools that are likely to aid the further investigation of this important subject. Here, we discuss the current *in vitro* and *in vivo* technologies that exist to enable study of female germline development and mtDNA transmission.

## *In vitro* models to investigate germline mtDNA transmission

It is only recently that induced pluripotent stem (iPS) cells derived from patients carrying heteroplasmic mtDNA variants have been used to understand the tissue specificity of mitochondrial diseases and to study the impact of the heteroplasmy upon cell fate (Cherry *et al*. 2013; Folmes *et al*. 2013; Hamalainen *et al*. 2013). These iPS‐derived cell models represent a potential tool to investigate the underlying disease mechanisms, but also to screen for new therapeutic drugs in a tissue‐specific manner (Hatakeyama & Goto 2016). This is possibly due to our comprehensive understanding of the molecular pathways involved in the differentiation of the iPS cells into different cell lineages. Such mechanisms, although already well established in iPS cells, have also been intensively studied in the context of PGC specification in recent years.

The PGC are specified from the proximal epiblast by BMP signaling from the extra‐embryonic tissues. This was first identified in homozygous BMP4 knockout mice, which do not develop and PGC, and a similar, but less drastic, phenotype was also observed in BMP8b null mice (Lawson *et al*. 1999; Ying *et al*. 2000). BMP signaling activates Blimp‐1, a key transcriptional regulator that is first expressed at E6.25 in mouse embryos. When Blimp1 expression is disrupted, the number of founder PGC drops from 40 to 20 and they no longer migrate to the genital ridge, where the future gonads will be formed (Ohinata *et al*. 2005). BMP signaling also activates expression of Prdm14, followed by Tcfap2c, which encodes the transcription factor AP2γ. Together Blimp1, Prdm14 and AP2γ control PGC specification and are able to rescue this process when expressed in the absence of BMP signaling (Magnusdottir *et al*. 2013). Further factors involved in PGC development were identified by Saitou *et al*. (2002) in a screen for PGC‐specific genes. They identified FGF‐8, a gene that is expressed in the early stages of PGC specification, and Stella, whose expression is germ cell‐specific at E7.25 and continues to be expressed in migrating PGC. This detailed understanding of PGC specification has enabled the *in vitro* induction of mouse and human ES cells, using growth factors including BMP4, BMP8 and bFGF, to produce primordial germ cell‐like cells (PGCLC), which recapitulate the gene expression profile of *in vivo* PGC (Hayashi & Saitou 2013; Sugawa *et al*. 2015). As proof of the robustness of this *in vitro* model, Hayashi *et al*. aggregated PGCLC with gonadal cells, which were then into mice depleted of endogenous PGC. These mice were subsequently bred and produced healthy offspring (Hayashi *et al*. 2012). Similarly, Hikabe *et al*. have reconstituted *in vitro* the entire cycle of mouse female germline. Using mouse ES cells, they were able to generate fully mature oocytes that could also give rise to healthy offspring when fertilized and transferred to surrogate mothers (Hikabe *et al*. 2016).

In the same way that iPS cells have expanded our understanding of the tissue specificity of mitochondrial diseases, *in vitro* modeling using PGCLC or mature oocytes derived from mouse and human ES cells are likely to represent a malleable tool to study the precise timing and mechanisms underpinning the transmission of mtDNA variants during oogenesis.

## Mouse models for *in vivo* study of mtDNA transmission

In addition to the use of *in vitro* PGCLC, there are also a number mouse models that allow *in vivo* examination of mtDNA inheritance, and several of these have already been discussed. A number of groups have generated artificial heteroplasmies by mixing mtDNA from different mouse strains. This approach has a number of drawbacks: first, unlike pathogenic variants in human mtDNA caused by single point mutations, the two mtDNA sequences in conplastic mice often contain a large number of nucleotide differences (e.g. 91 polymorphisms exist between the NZB and 129S6 strains, including 15 non‐synonymous variants [Sharpley *et al*. 2012]). This makes dissection of any selective mechanisms difficult, as multiple different factors may contribute to the dynamics of segregation in these animals. Also, in many cases, these heteroplasmies are not reported to be pathogenic (Jenuth *et al*. 1996; Meirelles & Smith 1997), and therefore may not be subject to purifying selection at all. Finally, introduction of a “foreign” mtDNA invariably leads to a nuclear/mtDNA mismatch within the cells, a situation that is exacerbated if two mtDNA sequences are backcrossed onto the nuclear background of a third strain (Sharpley *et al*. 2012). Because nuclear/mtDNA mismatching in conplastic mice is known to impact upon OXPHOS function and influence health and longevity (Latorre‐Pellicer *et al*. 2016), caution should be taken when interpreting results from these studies, as there is likely to be overlap between the effects of heteroplasmy and the mismatch with the nuclear genome.

The development of the *PolgA exo* ^−^ mtDNA mutator mouse, which acquires de novo heteroplasmic mtDNA mutations due to an inactivating mutation in the proof‐reading subunit of the mtDNA polymerase gamma (Trifunovic *et al*. 2004), has resulted in a much more tractable method for generating mice with genuine heteroplasmies. However, the rapid and random introduction of mutations into the mtDNA has made studying the inheritance of individual mtDNA mutations using this model challenging. By backcrossing *PolgA exo* ^−^ females with wild‐type males, Freyer *et al*. (2012) were able to generate a strain carrying just two mtDNA mutations: a homoplasmic substitution, m.5245T>C, in the tRNA^Cys^ gene and a heteroplasmic deletion, m.3875delC, in the tRNA^Met^ gene. Although these mice appeared phenotypically normal, a compensatory transcriptional response was seen in tissues carrying high levels of the tRNA^Met^ mutant, suggesting that this heteroplasmy was impacting mitochondrial function (Freyer *et al*. 2012). More recently, Kauppila *et al*. (2016) used a similar approach to obtain a strain carrying a single heteroplasmic mutation at m.5024C>T in the tRNA^Ala^ gene. Crucially, these animals develop cardiomyopathy and the mutation appears to be selected against in mitotic tissues, making this perhaps the best model currently available for studying inheritance of pathogenic mtDNA mutations in the mouse germline.

A number of tools exist to aid study of the mouse germline, allowing effective identification and isolation of PGC from their induction in the proximal epiblast at embryonic day (E)6.25 (Ohinata *et al*. 2005) through to oogenesis in late‐stage embryos. Staining of embryonic cells for PGC markers, such as tissue non‐specific alkaline phosphatase (Ginsburg *et al*. 1990), Stella (Dppa3) (Saitou *et al*. 2002) and Blimp1 (Prdm1) (Ohinata *et al*. 2005) allows efficient identification of PGC during early embryonic development; however, use of such stains and antibodies requires fixed tissues, limiting this approach to non‐living material. To enable more versatile studies in live cells, Payer *et al*. (2006) generated transgenic mice expressing a GFP‐tagged version of the PGC‐specific marker Stella, which allows non‐invasive identification of PGC from E7.5 onwards. Ohinata *et al*. (2008) subsequently built upon this model, creating a double‐transgenic reporter mouse expressing enhanced cyan fluorescent protein (ECFP)‐tagged Stella and mVenus‐tagged Blimp1. In this strain, Blimp1‐mVenus expression is seen from E7.5, with Stella‐ECFP detectable from E8.5, allowing for robust identification of double‐positive PGC. Crossing transgenic PGC reporter mice with females carrying mtDNA mutations, such as the m.5024C>T tRNA^Ala^ mutant, should enable detailed investigation of the dynamics involved in germline transmission of pathogenic mtDNA heteroplasmies and has the potential to dramatically expand our knowledge of this pivotal process.

## Studying the human germline *in vivo*

*In vivo* study of human germline development is incredibly challenging and fraught with ethical and technical difficulties. Whilst some data is available from oocytes and early embryos of patients carrying pathogenic heteroplasmies (Blok *et al*. 1997; Brown *et al*. 2001; Monnot *et al*. 2011), successful isolation of primary PGC from human embryos has not been reported. However, recently developed flow cytometry‐based protocols have been developed allowing the isolation of human PGC from Carnegie stage 12 onwards (Tang *et al*. 2015; Floros *et al*. 2018), representing a significant advance in our ability to investigate development of the human germline.

## Conclusions and perspectives

In recent years, huge advances have been made in the field of inherited mitochondrial disease, with a number of therapeutic approaches, such as pre‐implantation genetic screening (Smeets *et al*. 2015) and mitochondrial replacement therapy (Wolf *et al*. 2015), offering hope to families affected by these devastating conditions. However, despite the large number of studies aimed at understanding how heteroplasmic mtDNA mutations are transmitted through the germline, many of the mechanisms involved remain elusive, hampering efforts to develop more effective treatment and prevention strategies. The recent development of novel model systems and *in vivo* techniques for detailed investigation of germline development, both in humans and other mammals, promises to begin shedding light on some of the key unanswered questions that remain regarding mtDNA transmission, and will hopefully lead to tangible progress in the ongoing fight against mitochondrial disease.
